# *Pseudomonas aeruginosa* and *Burkholderia cepacia* complex co-infection in green nail syndrome: a case report

**DOI:** 10.3389/fmed.2026.1751352

**Published:** 2026-02-23

**Authors:** Ao Zhang, Haixia Kuang, Fan Chang, Hongxia Li, Zhuokun Ran, Xinyu Lin

**Affiliations:** 1 Department of Dermatology, Affiliated Hospital of Southwest Medical University, Luzhou, China; 2 Department of Dermatology, Yestar Plastic Surgery Hospital, Chengdu, China; 3 Department of Laboratory Medicine, Sichuan Provincial People's Hospital, University of Electronic Science and Technology of China, Chengdu, China; 4 Department of Clinical Medicine, North Sichuan Medical College, Nanchong, China; 5 Department of Dermatology, Sichuan Provincial People's Hospital, University of Electronic Science and Technology of China, Chengdu, China

**Keywords:** antimicrobial therapy, *Burkholderia cepacia* complex, case report, co-infection, diagnosis, green nail syndrome, *Pseudomonas aeruginosa*

## Abstract

Green nail syndrome (GNS) is classically characterized by a triad of green nail discoloration, paronychia, and onycholysis. Although *Pseudomonas aeruginosa* (*P. aeruginosa*) is the primary etiologic agent, infections involving co-pathogens are uncommon and pose considerable diagnostic and therapeutic challenges due to the absence of standardized management guidelines. We herein report a rare case of GNS caused by a co-infection with *P. aeruginosa* and the *Burkholderia cepacia* complex (BCC) in a woman with a history of manicures and frequent domestic exposure to water and detergents. The diagnosis was confirmed through bacterial culture and antimicrobial susceptibility testing. Targeted therapy involving a combination of oral and topical levofloxacin, guided by the antimicrobial susceptibility profile, resulted in the successful resolution of the infection. This case underscores the critical importance of microbiological identification for guiding effective treatment of non-classical GNS and demonstrates the efficacy of tailored antibiotic regimens in managing complex co-infections.

## Introduction

GNS, also known as chloronychia or Goldman-Fox syndrome, is a rare infection characterized by green discoloration of the nail plate; proximal paronychia and distal onycholysis often coexist (1). The primary causative agent is *Pseudomonas aeruginosa* (*P. aeruginosa*), which produces the aeruginous pigment and the aeruginous fluorophore, resulting in dark green to blue-green nail discoloration (2). The pathogenesis typically requires a compromised nail barrier, often associated with predisposing factors including chronic water exposure, manicures, trauma, or underlying nail conditions like onychomycosis (3).

While the diagnosis of GNS is primarily clinical (4), the condition can be challenging to manage due to the lack of standardized treatment guidelines and the potential for refractory courses or co-infections. Although *P. aeruginosa* is the predominant pathogen, infections involving other bacteria are rarely reported, and their optimal management remains poorly defined. *Burkholderia cepacia* complex (BCC), in particular, is an uncommon cause of skin and soft tissue infections (5), and its role in GNS has not been previously established.

We herein report a novel case of GNS resulting from a co-infection with *P. aeruginosa* and BCC. To our knowledge, this represents the first documented instance of such a co-infection in GNS. This case highlights the critical importance of microbiological culture for accurate pathogen identification and demonstrates the successful application of susceptibility-directed therapy in managing this complex presentation.

## Case description

A 37-year-old woman presented with green discoloration of the nail plates on her right fourth and fifth fingers, which had been present for over 10 days. The discoloration had initially appeared over 10 days prior and had progressively expanded and intensified. The patient reported no associated pain, pruritus, or discharge and had not sought any treatment prior to presentation. The remainder of the fingernails and toenails were unremarkable. Her history was significant for manicures preceding symptom onset and frequent exposure to water and detergents during domestic activities. She denied any history of local trauma. Her past medical history was otherwise unremarkable.

**Figure 1:**
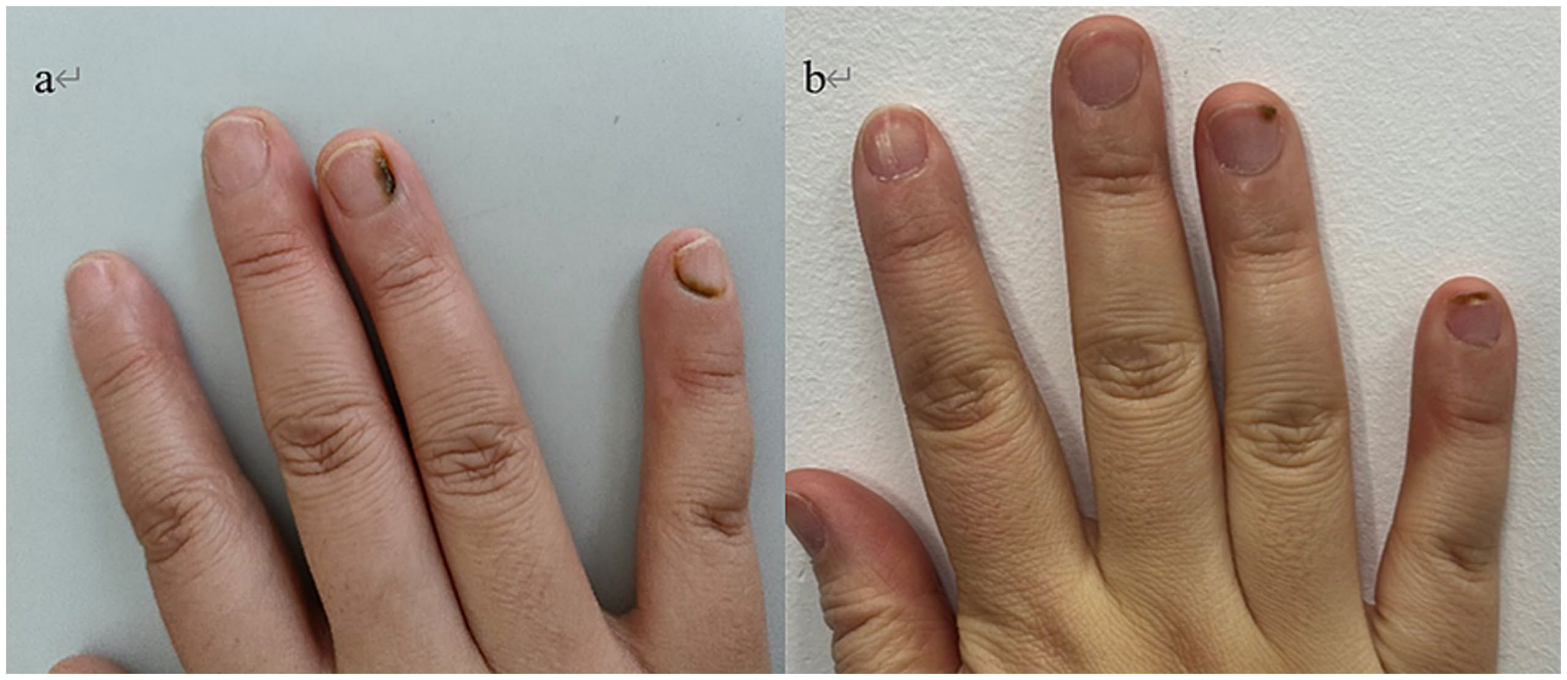
**(a)** Nail plate of a patient with trauma-related GNS at initial visit. **(b)** Lesion resolution after 3 months of combined oral and topical antibiotics.

Physical examination revealed no abnormalities on systemic assessment. Dermatological examination of the right fourth finger revealed a dark green discoloration of the nail plate, which extended to the lateral margins and involved the lunula. The proximal nail plate of the right fifth finger showed dark green discoloration. No significant onycholysis was observed. The perionychium of both affected nails showed no evidence of erythema, edema, or tenderness. The remaining fingernails and toenails were unremarkable (Figure 1a).

**Figure 2:**
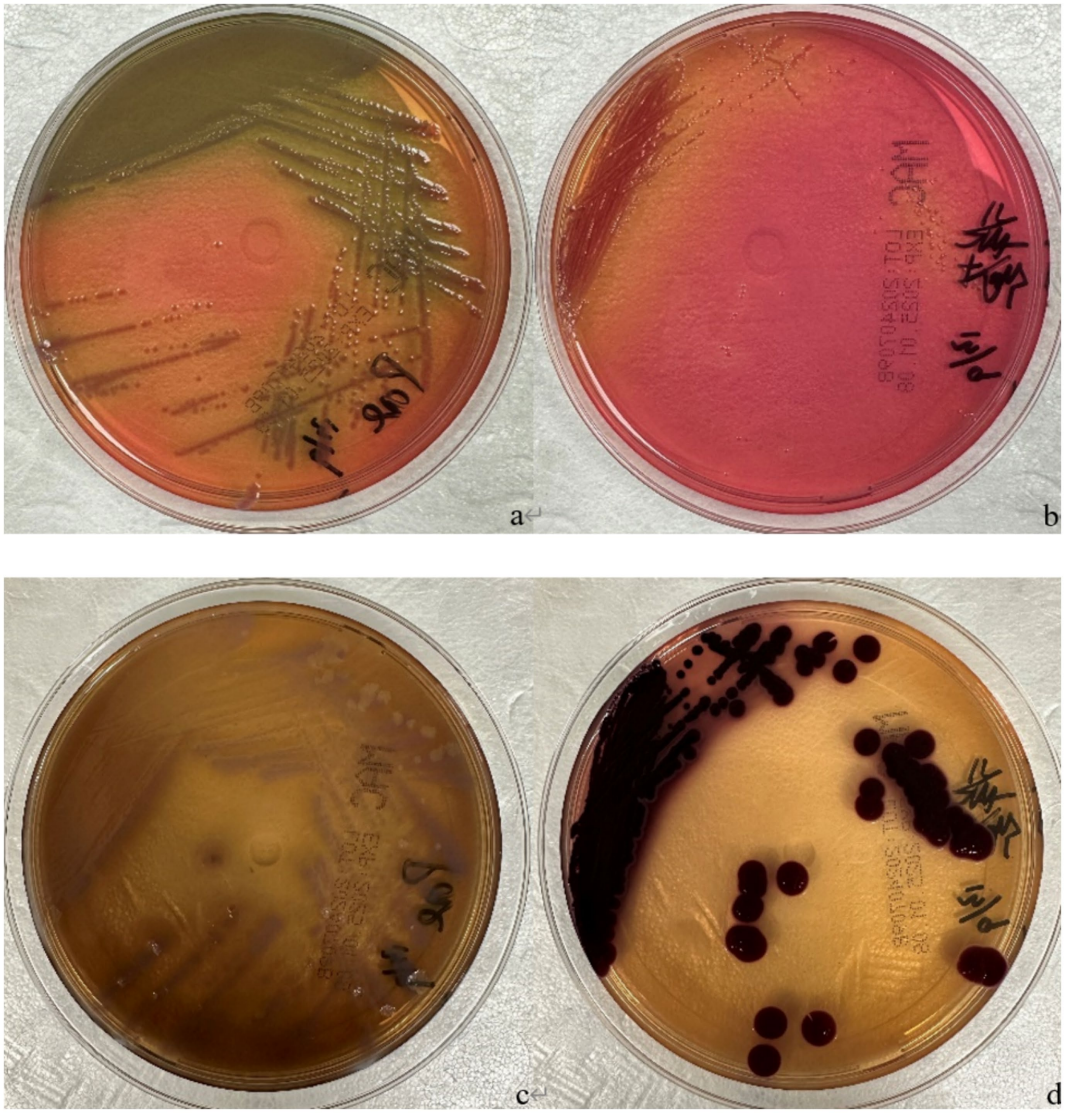
**(a)** *Pseudomonas aeruginosa*, MacConkey agar, incubated at 35 °C for 24 h; **(b)** *Burkholderia cepacia* complex, MacConkey agar, incubated at 35 °C for 24 h; **(c)** *Pseudomonas aeruginosa*, MacConkey agar, incubated at 35 °C for 6 days; **(d)** *Burkholderia cepacia* complex, MacConkey agar, incubated at 35 °C for 6 days.

Microbiological and dermoscopic investigations were performed. Direct microscopic examination with potassium hydroxide (KOH) preparation and direct immunofluorescence microscopy for fungi of the affected nails were both negative for fungal elements. Fungal culture on Sabouraud dextrose agar yielded no growth after 2 weeks of incubation. Conversely, bacterial culture of subungual debris on blood and MacConkey agar isolated *P. aeruginosa* and BCC (Figure 2). To minimize the risk of contamination from transient environmental flora or laboratory artifacts, a second subungual specimen was collected and cultured under identical conditions 1 week after the initial positive culture result. Both independent samples consistently yielded *P. aeruginosa* and bacteria belonging to the *Burkholderia* genus. The sampling procedure was strictly standardized to ensure specimen quality. On both occasions, sterile surgical blades were used to collect debris from the deep subungual space, minimizing the potential introduction of skin commensals or superficial contaminants. Furthermore, negative control cultures were processed in parallel throughout the testing period, all of which showed no bacterial growth, thereby ruling out the possibility of contamination from culture media or laboratory reagents.

**Figure 3:**
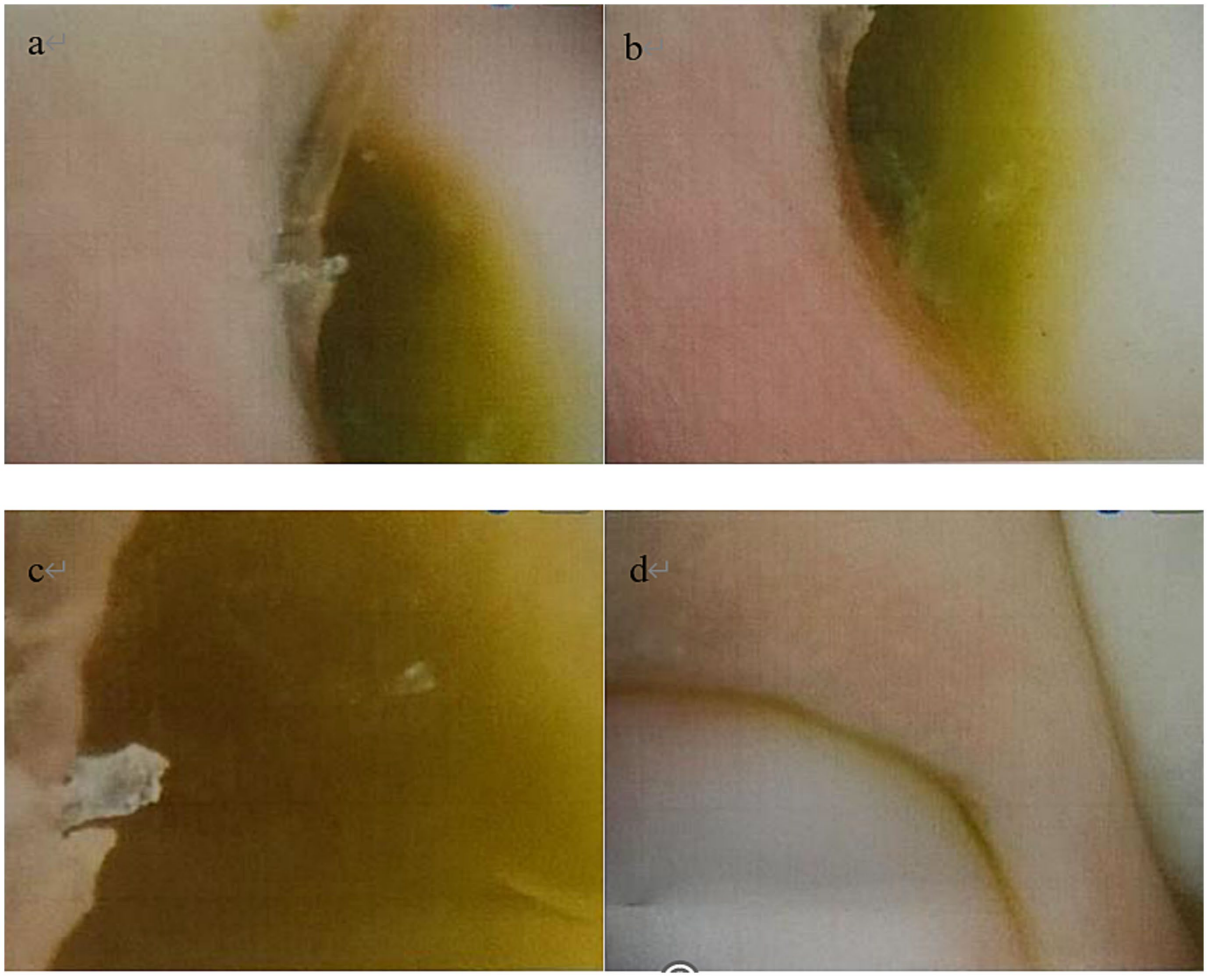
Dermoscopic examination of the right fourth finger revealed dark green pigmentation on the proximal nail plate **(a–c)**, with the coloration fading to a yellowish-green at the margins. The right fifth finger exhibited yellowish-green pigmentation on the proximal nail plate **(d)**.

Antimicrobial susceptibility testing, performed via the disk diffusion method, demonstrated sensitivity to multiple agents, including ceftazidime and levofloxacin (Figure 2). Dermoscopic examination revealed diffuse green pigmentation distributed along the nail plate, consistent with the clinical presentation of green nail syndrome (Figure 3).

The patient was then instructed to remove the gel nail polish and cease manicures. Based on the antimicrobial susceptibility profile (Table 1), a combined therapeutic regimen was initiated, consisting of oral levofloxacin (500 mg once daily for 1 month) and topical levofloxacin gel (applied twice daily to the affected nails for 3 months). After 3 months of continuous therapy, the green discoloration had progressively resolved from the proximal nail fold distally, and the lunula exhibited normal coloration (Figure 1b).

**Table 1:** Antimicrobial susceptibility testing results of the *Burkholderia cepacia* complex isolate.

Antimicrobial Agent	MIC (μg/mL)	Zone diameter (mm)	Interpretation	Interpretive criteria (S/R)
Levofloxacin	1		S	≤2 / ≥8
Ticarcillin/Clavulanic Acid	≥128		R	≤16 / ≥128
Ceftazidime	2		S	≤8 / ≥32
Minocycline		25	S	≥19 / ≤14
Trimethoprim/Sulfamethoxazole	≤20		S	≤40 / ≥80

MIC, Minimum inhibitory concentration. Susceptible (S), Intermediate (I), Resistant (R). Breakpoints were interpreted according to CLSI guidelines M100-S34 and M45 (3rd edition).

## Discussion

Green nail syndrome (GNS) denotes a triad of green discolouration of the nail plate, proximal paronychia, and distal nail separation, typically affecting only one or two nails (1). The primary causative agent is *P. aeruginosa*, which produces the aeruginous pigment and the aeruginous fluorophore, resulting in dark green to blue-green nail discoloration. *P. aeruginosa* is a Gram-negative, strictly aerobic, motile bacillus widely distributed in soil and vegetation, thriving particularly in aquatic environments. This pathogen exhibits relatively low virulence, typically causing disease only when local anatomical structures are compromised or immune function is deficient. It frequently causes secondary infections in immunocompromised individuals, colonizing sites such as burns, ulcers, and other moist skin surfaces (3). Susceptibility is heightened among patients receiving mechanical ventilation or those with underlying conditions, including metabolic disorders, malignancies, postoperative status, haematological disorders, or immunodeficiency. GNS often presents alongside other conditions, such as onychomycosis, psoriasis, or paronychia, particularly in moist environments. However, a green nail discoloration can also be caused by infections with other pathogens, including *Citrobacter*, *Enterococcus*, and *Fusarium* species (6). Therefore, accurate pathogen identification is crucial for both diagnosis and treatment.

The BCC comprises aerobic, Gram-negative, rod-shaped bacteria encompassing at least 24 phylogenetically related species (7). These bacteria are widely distributed in soil and water and can act as opportunistic pathogens in both humans and plants. In humans, BCC primarily causes opportunistic infections, with clinical manifestations ranging from asymptomatic carriage to severe conditions, such as lower respiratory tract infections in patients with cystic fibrosis (CF) and chronic granulomatous disease, or hematogenous infections in immunocompromised individuals (8). Although BCC species are uncommon agents of skin and soft tissue infections (SSTIs), we report this case due to the rarity of such infections in non-cystic fibrosis patients. To our knowledge, no concurrent infections involving *P. aeruginosa* and BCC in skin or skin appendages have been previously documented. To our knowledge, this case represents the first reported instance of a green nail syndrome (GNS) associated with co-infection by *P. aeruginosa* and BCC, successfully treated with oral and topical levofloxacin.

*Pseudomonas aeruginosa* alone is sufficient to cause classic Green Nail Syndrome. Therefore, the co-isolation of the BCC in this case reasonably calls into question its role in the disease process. However, several observations suggest its involvement beyond mere colonization: its repeated isolation from two independent cultures, its well-established status as an opportunistic pathogen, its recovery from a severe and chronic nail lesion, and its distinct antimicrobial susceptibility profile compared to *P. aeruginosa* (Tables 1, 2). Together, these factors indicate that BCC may function as an independent microbial entity capable of influencing the clinical course and treatment response. Both *P. aeruginosa* and BCC are known for their strong ability to form biofilms. We speculate that interactions within a potential polymicrobial biofilm at the infection site could promote bacterial coexistence, enhance tolerance to antimicrobial agents, and contribute to a more chronic and persistent infection. This may partly explain why an extended course of combination therapy was necessary in this case.

**Table 2:** Antimicrobial susceptibility testing results of the *Pseudomonas aeruginosa* isolate.

Antimicrobial agent	MIC (μg/mL)	Zone diameter (mm)	Interpretation	Interpretive criteria (S/R)
Levofloxacin	1		S	≤1 / ≥4
Ticarcillin/Clavulanic acid	16		S	≤16 / ≥128
Ceftazidime	2		S	≤8 / ≥32
Tetracycline	≥16		R	
Trimethoprim/Sulfamethoxazole	80		R	

MIC, Minimum inhibitory concentration. Susceptible (S), Intermediate (I), Resistant (R). Breakpoints were interpreted according to CLSI guidelines M100-S34 and M45 (3rd edition).

Of course, we must also objectively consider an alternative possibility. Given the patient’s history of nail plate damage from cosmetic manicures and prolonged exposure to water and detergents during housework, we cannot completely rule out that BCC was merely a secondary colonizer within the microenvironment created by *P. aeruginosa*. Clearly defining its role requires further investigation, such as *in vitro* biofilm formation assays, molecular epidemiological tracking of the isolates, or histological confirmation of tissue invasion.

The diagnosis of GNS is primarily clinical. However, one study (3) reported dermoscopic features in 32 patients with GNS, highlighting the utility of dermoscopy as an adjunctive diagnostic tool. While onychomycosis may present with a “Northern Lights” sign under dermoscopy, the specific finding of a “green Northern Lights” sign has been proposed to distinguish GNS from onychomycosis (6). In the present case, dermoscopic examination further confirmed the diagnosis.

Predisposing factors for GNS include frequent or prolonged water exposure, excessive use of soaps or detergents, nail trauma (e.g., from manicures), wearing tight footwear, nail-biting or picking, and underlying nail disorders such as psoriasis or onychomycosis. These factors can compromise the epidermal barrier of the nail, facilitating the invasion of pathogens like *P. aeruginosa* and the BCC. In this case, the patient reported a history of manicures involving trimming, filing, cuticle pushing, and gel polish application before symptom onset. Furthermore, frequent exposure to water and detergents during domestic activities likely contributed to initial nail plate damage and subsequent colonization. The subsequent application of gel polish may have created an occlusive environment that promoted a co-infection by these pathogens.

The optimal treatment regimen for GNS has not been standardized, underscoring the importance of obtaining bacterial cultures to identify non-*Pseudomonas* species and guide antibiotic selection based on susceptibility profiles. Both topical and systemic antibiotics may be used to eradicate the infection. In the present case, involving a co-infection with *P. aeruginosa* and the BCC without established guidelines, targeted antibiotic therapy was essential. Antimicrobial susceptibility testing (Table 3) confirmed that both isolated pathogens, *P. aeruginosa* and the BCC, were susceptible to levofloxacin and ceftazidime. The therapeutic decision in this case was based on a comprehensive evaluation of clinical features and drug characteristics, ultimately leading to the establishment of a levofloxacin-based “short-term systemic therapy combined with long-term topical consolidation” regimen.

**Table 3:** Overlapping antimicrobial susceptibility profiles of *Burkholderia cepacia* complex and *Pseudomonas aeruginosa.*

Antimicrobial agent	*Burkholderia cepacia* complex	*Pseudomonas aeruginosa*	Shared susceptibility
Levofloxacin	S	S	Yes
Ticarcillin/Clavulanic Acid	R	S	No
Ceftazidime	S	S	Yes
Trimethoprim/Sulfamethoxazole	S	R	No

Susceptible (S), Intermediate (I), Resistant (R). Breakpoints were interpreted according to CLSI guidelines M100-S34 and M45 (3rd edition).

First, the infection involved the lunula, indicating a deep-seated lesion where topical therapy alone might be insufficient to achieve effective drug concentrations. Second, the co-isolation of two recognized strong biofilm-forming bacteria suggests the likely presence of a polymicrobial biofilm in the subungual space. Biofilms can significantly enhance bacterial resistance to antimicrobial agents and host immune defenses, contributing to chronic and refractory infection. Therefore, systemic therapy is necessary to achieve and maintain drug concentrations within the nail apparatus that are sufficient to penetrate potential biofilms. Furthermore, the progressively worsening clinical presentation supported the need for more aggressive intervention.

Between the two susceptible agents, levofloxacin was selected as the superior choice due to its unique pharmacological and formulation advantages. As a fluoroquinolone, levofloxacin offers extremely high oral bioavailability and excellent tissue penetration, enabling it to reach concentrations well above the minimum inhibitory concentration (MIC) in relatively avascular tissues like the nail bed. Crucially, its availability in commercial topical formulations allows for the implementation of a “systemic plus topical” strategy. This approach enables synergistic action against both deep and superficial foci of infection and is particularly advantageous for eradicating potential biofilms. In contrast, ceftazidime (a third-generation cephalosporin) is not orally absorbed and must be administered via intravenous or intramuscular injection. Moreover, no topical formulation is available, precluding such a combination strategy. For a patient with a chronic nail infection managed on an outpatient basis, injectable therapy would significantly reduce treatment adherence, increase healthcare costs, and introduce risks associated with invasive procedures. Consequently, levofloxacin demonstrates clear advantages in terms of treatment accessibility, patient convenience, and overall therapeutic potential.

While complete fingernail regrowth in adults typically requires 4 to 6 months, the goal of initial systemic therapy is to rapidly control active infection. Therefore, a 4-week course of systemic levofloxacin was prescribed as the initial evaluation period. This duration represents a commonly effective treatment course to ensure adequate suppression and clearance of pathogens during the critical early phase. The treatment plan included an assessment at this point to decide on potential extension or regimen modification based on clinical response (e.g., resolution of green discoloration) and patient tolerance. We are fully aware of the risks associated with prolonged systemic fluoroquinolone use, including tendinitis, tendon rupture, peripheral neuropathy, and central nervous system effects. After thorough discussion of the risks and benefits with the patient and assessment of renal function, this shortest effective course was chosen to balance efficacy against systemic risk. The patient was instructed to monitor closely for related adverse effects throughout the treatment period.

Consequently, a regimen of oral levofloxacin (0.5 g/day for 1 month) combined with topical levofloxacin gel (applied twice daily for 3 months) was initiated. By the end of the 4-week treatment course, a clear clinical improvement was observed in this case. Therefore, the systemic therapy was completed as planned, and the regimen was transitioned to continued maintenance with topical treatment. This strategy aimed to consolidate the therapeutic effect and prevent relapse through local drug administration. It thereby ensured treatment efficacy while minimizing systemic drug exposure, achieving a balance between therapeutic effectiveness and patient safety. After 3 months of continuous therapy, the yellow-green discoloration progressively resolved from the distal end, and the lunula demonstrated normal coloration.

This study has several limitations. First, as a single case report, it cannot establish causality or generalize the findings to a broader population. Second, although the presence of BCC was confirmed by standard biochemical methods, we did not use molecular techniques such as 16S rRNA gene sequencing or whole-genome sequencing to identify the exact species within the complex. Given the significant heterogeneity in pathogenicity, environmental reservoirs, and antibiotic resistance profiles among different BCC species, the lack of species-level identification limits a more detailed interpretation of its clinical significance and potential epidemiological origin. Finally, the hypothesis of a polymicrobial biofilm interaction between *P. aeruginosa* and BCC, while reasonable based on their known biology, remains speculative without direct experimental validation, for example through *in vitro* biofilm assays.

Despite these limitations, our observations offer valuable insights for clinical practice. For cases of atypical, refractory, or recurrent green nail syndrome, we recommend performing comprehensive microbial cultures that include both bacterial and fungal pathogens, with high vigilance for potential polymicrobial infections. When BCC is isolated, species-level molecular identification and corresponding antimicrobial susceptibility testing are advised to guide precise treatment decisions. From a research perspective, this case highlights the need to accumulate data from more similar cases and to conduct *in vitro* studies investigating potential synergistic or antagonistic interactions between *P. aeruginosa* and BCC within biofilms. In clinical work, the possibility of mixed infection should be considered when formulating treatment expectations and strategies, as it may influence the required duration of therapy and the choice of antimicrobial agents. In cases of initial treatment failure, repeated microbial sampling and culturing should be performed to inform strategy adjustment.

## Data Availability

The original contributions presented in the study are included in the article/supplementary material, further inquiries can be directed to the corresponding author.

## References

[1] Heymann WR. Appending the appendages: new perspectives on netherton syndrome and green nail syndrome. J Am Acad Dermatol. (2020) 83:735–6. doi: 10.1016/j.jaad.2020.06.087, 32599118

[2] Spernovasilis N Psichogiou M Poulakou G. Skin manifestations of *Pseudomonas aeruginosa* infections. Curr Opin Infect Dis. (2021) 34:72–9. doi: 10.1097/QCO.0000000000000717, 33492004

[3] Ohn J Hur K Park H Cho S Mun JH. Dermoscopic patterns of green nail syndrome. J Eur Acad Dermatol Venereol. (2021) 35:e464–6. doi: 10.1111/jdv.17224, 33724580

[4] Yu Q Wang Y Yang H Li W Yang L. Case report: novel use of the conventional method—chemical nail avulsion may be effective for treatment of green nail syndrome. Front Med (Lausanne). (2022) 9:991918. doi: 10.3389/fmed.2022.991918, 36091683 PMC9452826

[5] Paes Leme RC Chaves JRE Gonçalves LCS Alvim LC Almeida JRC Renó LDC. Diabetic foot infection caused by bacteria of the *Burkholderia cepacia* complex: report of an unusual case and a scoping literature review. Rev Inst Med Trop Sao Paulo. (2022) 64:e36. doi: 10.1590/S1678-9946202264036, 35674634 PMC9173686

[6] Dominguez-Santas M Diaz-Guimaraens B Jimenez-Cauhe J Suarez-Valle A. Dermoscopy of green nail syndrome: the "green aurora sign". Dermatol Pract Concept. (2021) 11:e2021093. doi: 10.5826/dpc.1104a93, 35024221 PMC8648419

[7] Sfeir MM. *Burkholderia cepacia* complex infections: more complex than the bacterium name suggest. J Infect. (2018) 77:166–70. doi: 10.1016/j.jinf.2018.07.006 30012345

[8] Tavares M Kozak M Balola A Sá-Correia I. *Burkholderia cepacia* complex bacteria: a feared contamination risk in water-based pharmaceutical products. Clin Microbiol Rev. (2020) 33:e00139–19. doi: 10.1128/CMR.00139-19, 32295766 PMC7194853

